# Allergic enterocolitis and protein-losing enteropathy as the presentations of manganese leak from an ingested disk battery: A case report

**DOI:** 10.1186/1752-1947-2-286

**Published:** 2008-08-27

**Authors:** Muhammad A Altaf, Praveen S Goday, Grzegorz Telega

**Affiliations:** 1 Division of Pediatric Gastroenterology and Nutrition, Department of Pediatrics, The Medical College of Wisconsin, Watertown Plank Road, Milwaukee, WI 53221, USA

## Abstract

**Introduction:**

Disk battery ingestions can lead to serious complications including airway or digestive tract perforation, blood vessel erosions, mediastinitis, and stricture formation.

**Case presentation:**

We report a 20-month-old Caucasian child who developed eosinophilic enterocolitis and subsequent protein-losing enteropathy from manganese that leaked from a lithium disk battery. The disk battery was impacted in her esophagus for 10 days resulting in battery corrosion. We postulate that this patient's symptoms were due to a manganese leak from the 'retained' disk battery; this resulted in an allergic response in her gut and protein-losing enteropathy. Her symptoms improved gradually over the next 2 weeks with conservative management.

**Conclusion:**

This is the first case report to highlight the potential complication of allergic enterocolitis and protein-losing enteropathy secondary to ingested manganese. Clinicians should be vigilant about this rare complication in managing patients with disk battery ingestions.

## Introduction

**Figure 1:**
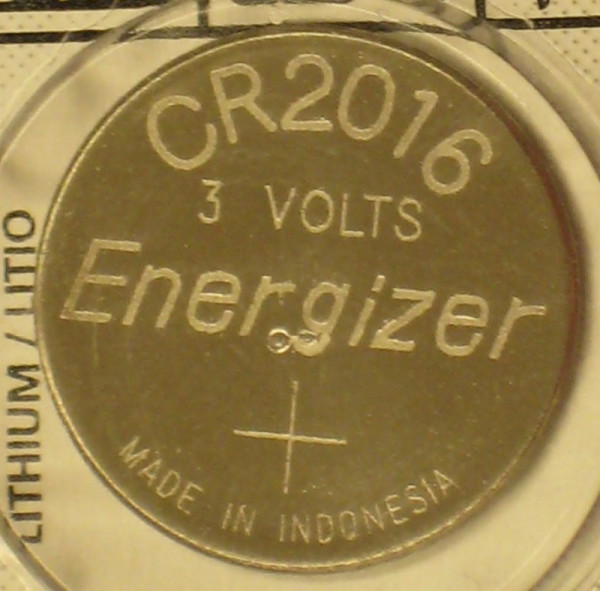
Lithium disk battery.

Lithium batteries are used in many portable consumer electronic devices (Fig. 1). The most common type of lithium cell used in consumer applications consists of lithium and manganese (Mn). Disk battery ingestions can lead to serious complications including aerodigestive tract perforation, vessel erosion, mediastinitis, and stricture formation [1]. Mercury batteries have been reported to cause more severe complications including acute poisoning [2], but none of the disk batteries have been reported to cause protein losing-enteropathy. We report a case in which the manganese in a lithium-manganese disk battery impacted in the esophagus presumably led to eosinophilic enterocolitis and severe protein-losing enteropathy.

## Case presentation

A 20-month-old Caucasian child presented with a 10-day history of vomiting and solid food refusal. Her chest X-ray showed a disk battery impaction in the upper esophagus. A corroded lithium-manganese battery was retrieved with a flexible laryngoscope 10 days after ingestion. The patient was transferred to our institution for further monitoring. Her physical examination and laboratory tests on admission were normal, except for an albumin of 2.7 g/dL (normal 3.8 to 5.4 g/dL) which had dropped from 4.3 g/dL on the day of battery removal. She had been on a regular diet until 10 days before admission. An esophagogram revealed no perforations.

**Figure 2:**
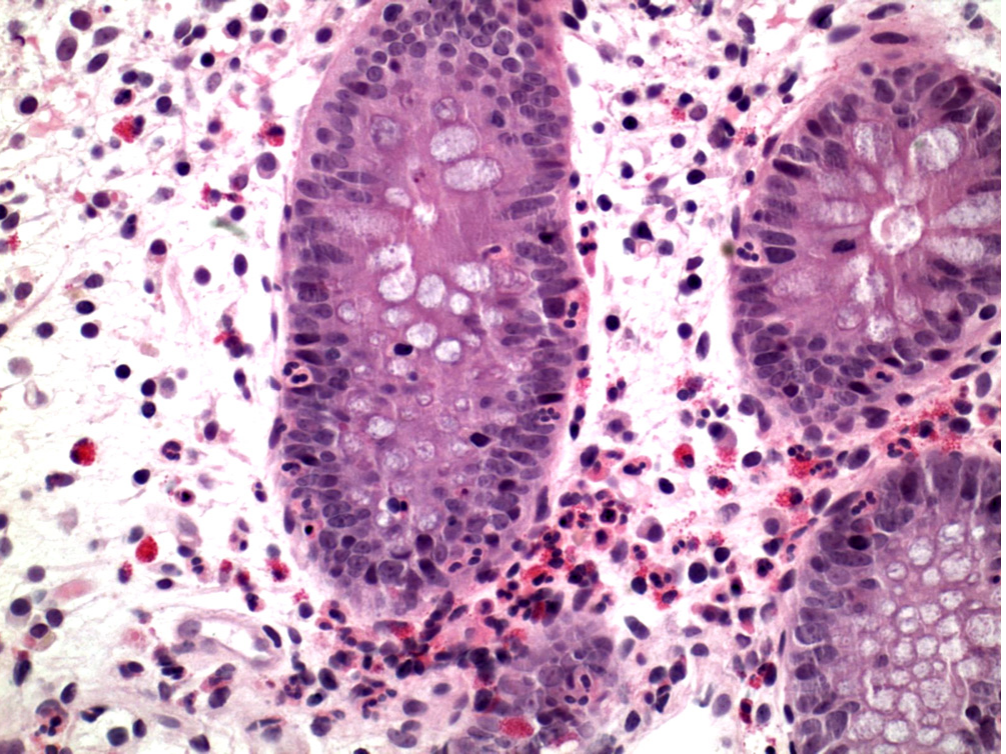
Colonoscopic biopsy showing eosinophilic infiltration of crypts in transverse colon.

A week following removal of the battery, she continued to refuse foods. An upper endoscopy was performed that revealed non-circumferential ulceration in the upper esophagus but no biopsies were performed at that time. Her stomach and duodenum were grossly normal at that time. A nasogastric (NG) tube was placed and feeding was started. Subsequently, the protein loss worsened and her serum albumin dropped to 1.1 g/dL (normal 3.8 to 5.4 g/dL). The urinalysis was normal and fecal alpha-1 antitrypsin level was 464 mg/g (normal <2 mg/g dry stool). A computed tomography (CT) scan of the abdomen, chest, and pelvis was performed because of persistent abdominal distention and feeding intolerance; this showed bilateral pleural effusions and moderate ascites. She had a normal echocardiogram and liver function tests. She was diagnosed with protein-losing enteropathy. Albumin 25% was started to maintain albumin at a level of >2 g/dL. A colonoscopy and repeat endoscopy were performed due to her protein-losing enteropathy; this showed complete healing of the previous esophageal ulceration, but with findings of diffuse enteritis and colitis. Small bowel biopsies were taken from the duodenum and terminal ileum. Histology revealed mild eosinophilic esophagitis and moderate eosinophilic enterocolitis (Fig. 2). In addition, the patient developed peripheral eosinophilia of 10.5% compared to 2.9% on admission. Her symptoms improved, and her albumin and eosinophilia normalized gradually over the next 2 weeks with conservative support and no steroids.

At 6 months follow-up, the patient has remained well, with normal albumin levels and no symptoms of protein-losing enteropathy. She developed an upper esophageal stricture that required recurrent dilatation and steroid injections. Her most recent endoscopic biopsies showed moderate eosinophils in the esophageal mucosa. We postulate that this patient's symptoms were due to a manganese leak from the 'retained' corroded disk battery before or during the process of removal from the esophagus; this caused an allergic response in her gut resulting in a protein-losing enteropathy.

## Discussion

The best known manifestations of chronic Mn exposure are neurological symptoms such as hypokinesia, rigidity and tremor that resemble Parkinson's disease [3]. Rarely, allergic responses have been described as well. Metal allergy to stainless steel wire containing Mn has been reported after coronary artery bypass grafting. A refractory pruritic erythematous wheal over the body with positive Mn patch testing and peripheral eosinophilia proved this to be a systemic allergic reaction to Mn [4]. Mn used in the manufacture of dental prosthesis has also been reported to cause contact dermatitis, manifested by diffuse oral edema, erythema and ulcerations; this was confirmed by positive patch testing [5,6].

Epidemiological studies have reported an acute impact of particulate Mn on the pulmonary system, including reversible decrement of pulmonary functions and increase in bronchial hyperreactivity [7,8]. In children, peak expiratory flow was decreased with a high concentration of Mn in the air, suggesting an obstructive allergic response rather than restrictive airway disease [9]. Exposure to high inhaled Mn concentrations has demonstrated an increased incidence of cough, rhinitis, bronchitis, and pneumonitis [10]. A study in rhesus monkeys documented subacute bronchiolitis and alveolar duct inflammation with lymphocytes, neutrophils, and a few eosinophils following inhalational exposure to Mn [11]. In general, serum or blood Mn does not serve as a reliable indicator of the total body burden of Mn because of its intracellular distribution and relatively short half-life [12].

We postulate that our patient developed an allergic enterocolitis and protein losing enteropathy in response to the Mn exposure in the gastrointestinal (GI) tract. The ingested battery was composed of lithium perchlorate and manganese dioxide. The possibility that some other component of the battery could have contributed to the pathogenesis cannot be ruled out, but in the literature, Mn is the only constituent that has been attributed to the allergic responses. This is supported by the previously suggested evidence that Mn can cause rhinitis, pneumonitis, and bronchial hyperreactivity. Manganese exposure from a cardiac stenting wire and dental prosthesis has also caused allergic symptoms with peripheral eosinophilia. Our patient's most recent esophageal biopsies suggest that she either had a baseline mild asymptomatic eosinophilic esophagitis that acutely worsened with exposure to Mn or the Mn was a trigger to her eosinophilic esophagitis.

## Conclusion

This case shows strong circumstantial evidence that the eosinophilic enterocolitis and protein-losing enteropathy were caused by the Mn leak from a retained disk battery; she was completely asymptomatic before battery ingestion with normal albumin levels and eosinophil counts before battery removal. Additionally, there was complete resolution without any treatment aside from removal of the Mn-containing disk battery. Clinicians should be vigilant about this rare complication while managing children with ingested disk batteries as symptoms might not appear immediately after battery removal.

## Abbreviations

CT: Computed Tomography; GI: Gastrointestinal; Mn: Manganese; NG: Nasogastric.

## Competing interests

The authors declare that they have no competing interests.

## Authors' contributions

All authors (MAA, PSG, GT) contributed in the management of the patient, writing of the manuscript and reviewing of the literature. All authors read and approved the final manuscript.

## Consent

Written informed consent was obtained from the parent for publication of this case report, as the child was a minor. A copy of the written consent is available for review by the Editor-in-Chief of this journal.

## References

[B1] Temple DM McNeese MC Hazards of battery ingestion Pediatrics 1983 71 1 100 103 6848955

[B2] Reilly DT Mercury battery ingestion Br Med J 1979 1 6167 859 435843 10.1136/bmj.1.6167.859 PMC1598483

[B3] Pal PK Samii A Calne DB Manganese neurotoxicity: a review of clinical features, imaging and pathology Neurotoxicology 1999 20 2–3 227 238 10385886

[B4] Takazawa K Ishikawa N Miyagawa H Yamamoto T Hariya A Dohi S Metal allergy to stainless steel wire after coronary artery bypass grafting J Artif Organs 2003 6 1 71 72 10.1007/s100470300011 14598128

[B5] Pardo J Rodriguez-Serna M De La Cuadra J Fortea JM Allergic contact stomatitis due to manganese in a dental prosthesis Contact Dermatitis 2004 50 1 41 10.1111/j.0105-1873.2004.00271b.x 15059103

[B6] Menezes LM Campos LC Quintao CC Bolognese AM Hypersensitivity to metals in orthodontics Am J Orthod Dentofacial Orthop 2004 126 1 58 64 10.1016/j.ajodo.2003.05.014 15224060

[B7] Boezen M Schouten J Rijcken B Vonk J Gerritsen J Zee S van der Hoek G Brunekreef B Postma D Peak expiratory flow variability, bronchial responsiveness, and susceptibility to ambient air pollution in adults Am J Respir Crit Care Med 1998 158 6 1848 1854 9847277 10.1164/ajrccm.158.6.9804072

[B8] Sharma M Kumar VN Katiyar SK Sharma R Shukla BP Sengupta B Effects of particulate air pollution on the respiratory health of subjects who live in three areas in Kanpur, India Arch Environ Health 2004 59 7 348 358 10.3200/AEOH.59.7.348-358 16241039

[B9] Hong YC Hwang SS Kim JH Lee KH Lee HJ Lee KH Yu SD Kim DS Metals in particulate pollutants affect peak expiratory flow of school children Environ Health Perspect 2007 115 3 430 434 17431494 10.1289/ehp.9531 PMC1849935

[B10] Roels H Lauwerys R Buchet JP Genet P Sarhan MJ Hanotiau I de Fays M Bernard A Stanescu D Epidemiological survey among workers exposed to manganese: effects on lung, central nervous system, and some biological indices Am J Ind Med 1987 11 3 307 327 10.1002/ajim.4700110308 3578289

[B11] Dorman DC Struve MF Gross EA Wong BA Howroyd PC Sub-chronic inhalation of high concentrations of manganese sulfate induces lower airway pathology in rhesus monkeys Respir Res 2005 6 121 10.1186/1465-9921-6-121 16242036 PMC1283983

[B12] Lu L Zhang LL Li GJ Guo W Liang W Zheng W Alteration of serum concentrations of manganese, iron, ferritin, and transferrin receptor following exposure to welding fumes among career welders Neurotoxicology 2005 26 2 257 265 10.1016/j.neuro.2004.09.001 15713346 PMC4002285

